# Special Issue “Cutaneous Lymphomas”

**DOI:** 10.3390/cancers15051481

**Published:** 2023-02-26

**Authors:** Marion Wobser, Matthias Goebeler

**Affiliations:** Department of Dermatology, Venereology and Allergology, University Hospital Würzburg, 97080 Würzburg, Germany

Cutaneous lymphomas comprise heterogeneous subtypes of hematological neoplasms that primarily manifest in the skin [1,2]. A close correlation of histological, immunophenotypical and clinical findings is mandatory in order to attribute the respective lymphoma subtype, especially owing to the fact that clinical prognosis—and consequently the appropriate therapeutic regimen—vary significantly between different entities.

During the last years, tremendous success in delineating the molecular pathogenesis of cutaneous lymphomas has been achieved. Especially with respect to the most common skin lymphomas, i.e., mycosis fungoides and Sézary syndrome, a better comprehension of the cell of origin [3], its molecular alterations [4] and the interaction with the tumor microenvironment [5] could translate into improved evaluation and development of novel therapeutic concepts [6]. Nevertheless, cutaneous lymphomas are rare tumor entities and, therefore, during the last years relatively few large randomized clinical trials have been conducted. Consecutively, collaborative data compilation within national or international registries as well as the evaluation of real-world data with respect to diagnosis, prognosis and treatment of cutaneous lymphomas are of paramount importance [7]. 

Taking this background into mind, this Special Issue, entitled “Cutaneous Lymphomas”, aimed to addressing all of these multifaceted issues within the field of cutaneous lymphomas. 

One focus of this Special Issue was to present epidemiological and real-world data on different subtypes of cutaneous lymphomas arising from collaborative national and international projects. Within this context, a comprehensive review and meta-analysis including more than 16,000 cases of different subtypes of cutaneous T-cell lymphomas is presented which describes key epidemiological features from a global perspective [8]. In addition, the article of K. Wojewoda and colleagues [9] summarizes epidemiological, diagnostic and clinical data on patients with mycosis fungoides in a monocentric national setting (Sweden), clearly underlining the importance of stage-adapted treatment as provided by national and international guidelines [10]. Of note, retrospective data from a multicenter chart review compiling entries between 2017–2018 delineated significant variations in treatment choice for relapsed/refractory (R/R) cutaneous T-cell lymphomas in a real-world setting in European countries [11]. In particular, the choice of chemotherapeutic protocols as well as applied combinatory approaches differed widely between the 27 contributing European centres with respect to first-, second- and third-line treatments as well as to R/R conditions. In this retrospective analysis, beyond interferon, retinoids and phototherapy, chemotherapy as a first-line treatment option turned out to be more frequently applied in the clinical routine when compared to previously published data that have delineated extracorporeal photopheresis, bexarotene, phototherapy and methotrexate as the main first-line treatment modalities [12]. Although novel treatment options were uncommonly used according to this retrospective chart review (that considered data collected between 2017 and 2018) [11], the authors conclude that a wider application of novel targeted therapies such as brentuximab vedotin and mogamulizumab might alter treatment patterns and outcomes in the future. 

Another focus of the Special Issue was to present novel insights into the molecular pathogenesis of different subtypes of cutaneous lymphomas. While previous investigations have delineated key molecular alterations as well as distinct immunophenotypes of the different cutaneous B-cell lymphomas subtypes, namely cutaneous marginal zone lymphoma [13,14] and cutaneous diffuse large B-cell lymphoma [15], up to now there had only limited data been available that addressed the molecular pathogenesis of cutaneous follicular B-cell lymphoma [16]. The article contributed by our research group [17] adds additional deep sequencing data to close this gap in elucidating the mutational landscape of cutaneous follicular B-cell lymphoma. Genetic alterations within 15 of the selected target genes were identified, with prevailing somatic mutations present in *TNFRSF14, CREBBP, STAT6* and *TP53* genes. While several of these molecular findings show overlap with genetic alterations in systemic follicular B-cell lymphoma, the presence of any of these mutations was not associated with biological behaviour of cutaneous B-cell lymphoma. Nevertheless, such molecular profiling might serve to facilitate discriminating cutaneous B-cell lymphoma from pseudolymphoma in the future.

Owing to the indolent behaviour of cutaneous follicular B-cell lymphoma, skin-directed treatment options are mainstays of care. Taking this into account, intralesional versus intravenous applications of rituximab were retrospectively assessed in a monocentric analysis of indolent cutaneous B-cell lymphomas [18]. Neither treatment response nor relapse rate showed statistically significant differences between either treatment regimen. In addition, potential adverse effects were amenable, indicating that intralesional administration of rituximab might be a feasible therapeutic option for indolent cutaneous B-cell lymphoma of a limited extent.

Taking translational aspects into account, two further articles and one comprehensive review add further data on the molecular pathogenesis of cutaneous T-cell lymphomas to this Special Issue. Although large-cell transformation (LCT) of mycosis fungoides has been associated with a higher risk of relapse and progression and, consequently, restricted prognosis [19], its molecular pathogenesis had not been elucidated yet. Novel data published herein demonstrate that the large-cell transition of mycosis fungoides is associated with a higher frequency of somatic mutations in cancer-associated genes [20]. In particular, the activation of RAS signaling together with epigenetic dysregulation may orchestrate the altered phenotype of LCT and provide the molecular basis for potentially adverse clinical behavior. F. Karagianni et al. evaluated the chick embryo chorioallantoic membrane model (CAM) assay as a preclinical in vivo model for cutaneous T-cell lymphoma that allows for pharmacological testing [21]. Combining the HDAC inhibitor resminostat with the JAK-inhibitor ruxolitinib displayed significant antitumor effects in established cell lines of mycosis fungoides and Sézary syndrome. Taking into account the presence of frequent alterations in JAK/STAT signalling as well as in epigenetic regulation in cutaneous T-cell lymphoma, such a combinatory therapeutic approach might be feasible for further evaluation in the clinical setting.

The biological behaviour of cutaneous T-cell lymphomas is not only determined by characteristics of the neoplastic cell itself—as exemplarily depicted above—but, more-over, also by its close interaction with the tumor microenvironment and by influences imparted by the skin microbiome [22]. To further account for this complexity, this Special Issue includes a review article that gives an overview on the role of the cutaneous microbiome for cutaneous T-cell lymphomas. Defective skin barriers and chronic antigen exposure by microbial components or superantigens have been determined to have a crucial role in stimulating the disease progression of mycosis fungoides and vice versa, indicating that antimicrobial strategies might hence be exploited for the treatment of therapeutic issues [23].

In summary, this Special Issue comprises informative research articles and reviews, written by international experts in the field, which pinpoint key milestones of current research within the field of cutaneous lymphomas. Moreover, open questions and future tasks are comprehensively discussed with the aim of tackling diagnostic and therapeutic challenges in the future. 

## References

[1] Willemze R., Cerroni L., Kempf W., Berti E., Facchetti F., Swerdlow S.H., Jaffe E.S. (2019). The 2018 update of the WHO-EORTC classification for primary cutaneous lymphomas. Blood.

[2] Dummer R., Vermeer M.H., Scarisbrick J.J., Kim Y.H., Stonesifer C., Tensen C.P., Geskin L.J., Quaglino P., Ramelyte E. (2021). Cutaneous T cell lymphoma. Nat. Rev. Dis. Prim..

[3] Campbell J.J., Clark R.A., Watanabe R., Kupper T.S. (2010). Sezary syndrome and mycosis fungoides arise from distinct T-cell subsets: A biologic rationale for their distinct clinical behaviors. Blood.

[4] Tensen C.P., Quint K.D., Vermeer M.H. (2022). Genetic and epigenetic insights into cutaneous T-cell lymphoma. Blood.

[5] Lindahl L.M., Willerslev-Olsen A., Gjerdrum L.M.R., Nielsen P.R., Blümel E., Rittig A.H., Celis P., Herpers B., Becker J.C., Stausbøl-Grøn B. (2019). Antibiotics inhibit tumor and disease activity in cutaneous T-cell lymphoma. Blood.

[6] Khodadoust M.S., Mou E., Kim Y.H. (2022). Integrating Novel Agents into the Treatment of Advanced Mycosis Fungoides and Sézary Syndrome. Blood.

[7] Scarisbrick J.J., Prince H.M., Vermeer M.H., Quaglino P., Horwitz S., Porcu P., Stadler R., Wood G.S., Beylot-Barry M., Pham-Ledard A. (2015). Cutaneous Lymphoma International Consortium Study of Outcome in Advanced Stages of Mycosis Fungoides and Sézary Syndrome: Effect of Specific Prognostic Markers on Survival and Development of a Prognostic Model. J. Clin. Oncol. Off. J. Am. Soc. Clin. Oncol..

[8] Dobos G., Pohrt A., Ram-Wolff C., Lebbé C., Bouaziz J.D., Battistella M., Bagot M., de Masson A. (2020). Epidemiology of Cutaneous T-Cell Lymphomas: A Systematic Review and Meta-Analysis of 16,953 Patients. Cancers.

[9] Wojewoda K., Gillstedt M., Englund H., Ali S., Lewerin C., Osmancevic A. (2022). Diagnostic Outcomes and Treatment Modalities in Patients with Mycosis Fungoides in West Sweden-A Retrospective Register-Based Study. Cancers.

[10] Trautinger F., Eder J., Assaf C., Bagot M., Cozzio A., Dummer R., Gniadecki R., Klemke C.D., Ortiz-Romero P.L., Papadavid E. (2017). European Organisation for Research and Treatment of Cancer consensus recommendations for the treatment of mycosis fungoides/Sézary syndrome—Update 2017. Eur. J. Cancer.

[11] Assaf C., Waser N., Bagot M., He M., Li T., Dalal M., Gavini F., Trinchese F., Zomas A., Little M. (2021). Contemporary Treatment Patterns and Response in Relapsed/Refractory Cutaneous T-Cell Lymphoma (CTCL) across Five European Countries. Cancers.

[12] Quaglino P., Maule M., Prince H.M., Porcu P., Horwitz S., Duvic M., Talpur R., Vermeer M., Bagot M., Guitart J. (2019). Global patterns of care in advanced stage mycosis fungoides/Sezary syndrome: A multicenter retrospective follow-up study from the Cutaneous Lymphoma International Consortium. Ann. Oncol..

[13] Maurus K., Appenzeller S., Roth S., Kuper J., Rost S., Meierjohann S., Arampatzi P., Goebeler M., Rosenwald A., Geissinger E. (2018). Panel Sequencing Shows Recurrent Genetic FAS Alterations in Primary Cutaneous Marginal Zone Lymphoma. J. Investig. Dermatol..

[14] Carlsen E.D., Swerdlow S.H., Cook J.R., Gibson S.E. (2019). Class-switched Primary Cutaneous Marginal Zone Lymphomas Are Frequently IgG4-positive and Have Features Distinct From IgM-positive Cases. Am. J. Surg. Pathol..

[15] Mareschal S., Pham-Ledard A., Viailly P.J., Dubois S., Bertrand P., Maingonnat C., Fontanilles M., Bohers E., Ruminy P., Tournier I. (2017). Identification of Somatic Mutations in Primary Cutaneous Diffuse Large B-Cell Lymphoma, Leg Type by Massive Parallel Sequencing. J. Investig. Dermatol..

[16] Barasch N.J.K., Liu Y.C., Ho J., Bailey N., Aggarwal N., Cook J.R., Swerdlow S.H. (2020). The molecular landscape and other distinctive features of primary cutaneous follicle center lymphoma. Hum. Pathol..

[17] Wobser M., Schummer P., Appenzeller S., Kneitz H., Roth S., Goebeler M., Geissinger E., Rosenwald A., Maurus K. (2022). Panel Sequencing of Primary Cutaneous B-Cell Lymphoma. Cancers.

[18] Menzer C., Rendon A., Hassel J.C. (2022). Treatment of Indolent Cutaneous B-Cell Lymphoma with Intralesional or Intravenous Rituximab. Cancers.

[19] Benner M.F., Jansen P.M., Vermeer M.H., Willemze R. (2012). Prognostic factors in transformed mycosis fungoides: A retrospective analysis of 100 cases. Blood.

[20] Wobser M., Roth S., Appenzeller S., Houben R., Schrama D., Goebeler M., Geissinger E., Rosenwald A., Maurus K. (2021). Targeted Deep Sequencing of Mycosis Fungoides Reveals Intracellular Signaling Pathways Associated with Aggressiveness and Large Cell Transformation. Cancers.

[21] Karagianni F., Piperi C., Casar B., de la Fuente-Vivas D., García-Gómez R., Lampadaki K., Pappa V., Papadavid E. (2022). Combination of Resminostat with Ruxolitinib Exerts Antitumor Effects in the Chick Embryo Chorioallantoic Membrane Model for Cutaneous T Cell Lymphoma. Cancers.

[22] Jost M., Wehkamp U. (2022). The Skin Microbiome and Influencing Elements in Cutaneous T-Cell Lymphomas. Cancers.

[23] Lindahl L.M., Iversen L., Ødum N., Kilian M. (2022). Staphylococcus aureus and Antibiotics in Cutaneous T-Cell Lymphoma. Dermatology.

